# Documenting Community Health Worker Compensation Schemes and Their Perceived Effectiveness in Seven sub-Saharan African Countries: A Qualitative Study

**DOI:** 10.9745/GHSP-D-24-00008

**Published:** 2024-06-27

**Authors:** Alyssa L. Davis, Lola Flomen, Jehan Ahmed, Djibrilla Maiga Arouna, Amos Asiedu, Maman Bacharou Badamassi, Ousmane Badolo, Moumouni Bonkoungou, Ciro Franco, Zachariah Jezman, Victoria Kalota, Beh Kamate, Daniel Koko, John Munthali, Raphael Ntumy, Patrick Sichalwe, Oumar Yattara

**Affiliations:** a Consultant, PMI Impact Malaria, Washington, DC, USA.; b Consultant, Population Services International, Washington, DC, USA.; c PMI Impact Malaria, Washington, DC, USA.; d PMI Impact Malaria, Niamey, Niger.; e PMI Impact Malaria, Accra, Ghana.; f PMI Impact Malaria, Ouagadougou, Burkina Faso.; g PMI Impact Malaria, Lilongwe, Malawi.; h PMI Impact Malaria, Lusaka, Zambia.; i PMI Impact Malaria, Bamako, Mali.

## Abstract

**Introduction:**

Community health worker (CHW) incentives and remuneration are core issues affecting the performance of CHWs and health programs. There is limited documentation on the implementation details of CHW financial compensation schemes used in sub-Saharan African countries, including their mechanisms of delivery and effectiveness. We aimed to document CHW financial compensation schemes and understand CHW, government, and other stakeholder perceptions of their effectiveness.

**Methods:**

A total of 68 semistructured interviews were conducted with a range of purposefully selected key informants in 7 countries: Benin, Burkina Faso, Ghana, Malawi, Mali, Niger, and Zambia. Thematic analysis of coded interview data was conducted, and relevant country documentation was reviewed, including any documents referenced by key informants, to provide contextual background for qualitative interpretation.

**Results:**

Key informants described compensation schemes as effective when payments are regular, distributions are consistent, and amounts are sufficient to support health worker performance and continuity of service delivery. CHW compensation schemes associated with an employed worker status and government payroll mechanisms were most often perceived as effective by stakeholders. Compensation schemes associated with a volunteer status were found to vary widely in their delivery mechanisms (e.g., cash or mobile phone distribution) and were perceived as less effective. Lessons learned in implementing CHW compensation schemes involved the need for government leadership, ministerial coordination, community engagement, partner harmonization, and realistic transitional financing plans.

**Conclusion:**

Policymakers should consider these findings in designing compensation schemes for CHWs engaged in routine, continuous health service delivery within the context of their country’s health service delivery model. Systematic documentation of the tasks and time commitment of volunteer status CHWs could support more recognition of their health system contributions and better determination of commensurate compensation as recommended by the 2018 World Health Organization *Guidelines on Health Policy and System Support to Optimize Community Health Worker Programs.*

## Abstract (precis)

Systematic documentation of the tasks and time commitment of CHWs, particularly those with a volunteer status, could support more recognition of their health system contributions and better determination of commensurate compensation.

## Teaser

**Key Findings:**

- Financial compensation schemes and service delivery models varied greatly across Benin, Burkina Faso, Ghana, Malawi, Mali, Niger, and Zambia, with each country engaging community health workers (CHWs) with different responsibilities, catchment areas, and relationships to health facilities.
- The majority of the countries used a dual-cadre service delivery model in which CHWs who were at least partially based at facilities were considered to be full-time employees and paid a salary, while CHWs who were based in communities were considered part-time volunteers and given a stipend or activity-based incentive.
- Key informants described several challenges with compensation schemes for volunteer status CHWs, including irregular payments, inconsistent distribution, and insufficient amount of compensation, which led to demotivation and attrition of CHWs and, in turn, negatively affected health service continuity.

**Key Implications:**

- Policymakers should consider these findings in the design of compensation schemes for CHWs engaged in routine, continuous health service delivery within the context of their country’s health service delivery model.
- Greater sensitization of the 2018 World Health Organization recommendation on CHW compensation, along with practical guidance on how to assess CHW responsibilities, workload, and training, may be needed to support the practical application of this global normative guidance at the country level.

## INTRODUCTION

Community health worker (CHW) incentives and remuneration are core issues that affect the performance of individual CHWs and overall health programs.^1^ In low- and lower-middle-income countries, approximately half of CHWs providing services on behalf of governments receive a salary, while the other half receive payments per day or activity, in-kind benefits, or no benefits at all.^2^ When considering all types of CHWs (not only those providing services on behalf of the government), it is estimated that as many as 85% of CHWs on the African continent do not receive a salary for their work.^3^

In 2018, the World Health Organization (WHO) released *Guidelines on Health Policy and System Support to Optimize Community Health Worker Programs*, which outlined a 2-part recommendation for the compensation of CHWs. The first part recommends “remunerating CHWs for their work with a financial package commensurate with job demands, complexity, number of hours, training, and roles that they undertake.”^4^ The second part advises “not paying CHWs exclusively or predominantly according to performance-based incentives.”^4^ While literature indicates that performance-based financial incentives can motivate CHWs when they have appropriate control over the incentivized task, the Guideline Development Group expressed concern based on existing review evidence that nonincentivized tasks may be deprioritized in favor of incentivized tasks.^5^ These recommendations are based on evidence that incentives—both financial and nonfinancial—can positively impact CHW performance. Additionally, the Guideline Development Group specifically noted that nonfinancial incentives should “not be seen as a substitute for the provision of a financial package, and conversely, that the provision of a financial package should not be seen as a substitute for nonfinancial incentives.”

Beyond broad categorizations (e.g., salaried vs. volunteer), there is limited documentation on the details of CHW financial compensation schemes used across countries in sub-Saharan Africa, including their mechanisms of delivery and effectiveness. To contribute to filling the practical information gap on CHW financial compensation schemes across countries in sub-Saharan Africa and document challenges and lessons learned from country experiences in implementing financial compensation schemes, the U.S. President’s Malaria Initiative (PMI) commissioned a study on financial compensation schemes involving CHWs engaged in the provision of integrated community case management (iCCM) services. iCCM is a strategy to extend case management of key childhood illness beyond health facilities so that more children have access to lifesaving treatments.^6^ We aimed to document CHW financial compensation schemes and stakeholder perceptions of their effectiveness.

There is limited documentation on the details of CHW financial compensation schemes used across countries in sub-Saharan Africa.

## METHODS

### Country Selection

This study was conducted by PMI’s Impact Malaria project. The study included 7 countries: Benin, Burkina Faso, Ghana, Malawi, Mali, Niger, and Zambia. These countries were selected from among the list of PMI partner countries in Africa, considering the following criteria: inclusion of language on financial compensation within the country’s community health policy; implementation of some type of financial compensation for CHWs providing iCCM services; geographic and linguistic diversity; and preference for inclusion of countries where PMI had less existing documentation of their community health programs.

### Study Objectives

The objectives of this study were to: (1) document the financial compensation schemes implemented for CHWs providing iCCM services; (2) gather stakeholder perspectives about the effectiveness of the CHW financial compensation schemes implemented; (3) identify challenges and lessons learned from stakeholder experiences implementing CHW financial compensation schemes.

### Key Informant Selection

Key informants engaged in supporting community health and iCCM programs at a national level were identified by the research team across all 7 countries. Additionally, key informants working at a subnational level were identified across Mali, Malawi, and Niger. National-level key informants included representatives from national malaria control programs, child health programs, and any ministry of health (MOH) department responsible for community health or iCCM services more specifically, as well as donors and implementing partners, such as community-based organizations, civil society organizations, nongovernmental organizations (NGOs) and United Nations (UN) agencies engaged in supporting CHWs providing iCCM services. At the subnational level, 1–2 health service administration areas per country were selected from among those where the PMI Impact Malaria project had an operational presence for purposeful selection of key informants, specifically MOH administrators, health facility staff, and CHWs. An initial key informant list was developed by the research team’s country focal points (i.e., PMI Impact Malaria project staff engaged in community health and iCCM services within the country for several years), and additional key informants were identified based on referrals by those initially contacted.

A total of 68 key informant interviews were conducted across the 7 countries (Table 1). Over half of the key informants were government staff, and all were individuals with many years of experience working within their country’s health sector, often in multiple roles over the course of their careers (e.g., different positions within the MOH or transitions between government, donor, NGO, or UN positions). Key informant categories were determined based on the organizational affiliation and the role of the individual at the time they were interviewed. As this study focused on CHWs who provide iCCM services, only CHWs responsible for providing iCCM services were interviewed (i.e., health surveillance assistants in Malawi, agents de santé communautaire in Mali, and relais communautaire in Niger). Table 2 provides details on all types of CHWs across the 7 countries.

**TABLE 1.** Key Informants Interviewed Across Seven sub-Saharan African Countries

	**Benin**	**Burkina Faso**	**Ghana**	**Malawi**	**Mali**	**Niger**	**Zambia**	**Total**
Government	2	3	1	5	10	13	3	37
Nongovernmental organization	2	0	1	2	2	0	3	10
United Nations agencies	1	0	0	0	1	1	0	3
Donor	0	0	1	1	0	1	1	4
Private-sector organization	0	1	0	0	0	0	0	1
Community health worker	0	0	0	8	2	3	0	13
Total	5	4	3	16	15	18	7	68

**TABLE 2.** Overview of Community Health Worker Financial Compensation Schemes in Seven sub-Saharan African Countries

	**Benin**		**Burkina Faso**	**Ghana**		**Malawi**		**Mali**	**Niger**		**Zambia**	
	**Agents de Santé Communautaire Qualifié**	**Relais Communautaire**	**Agents de Santé à Base Communautaire**	**Community Health Officer**	**Community Based Volunteer**	**Health Surveillance Assistant**	**Community Health Volunteer**	**Agents de Santé Communautaire**	**Agents de Santé Communautaire**	**Relais Communautaire**	**Community Health Assistant**	**Community Based Volunteer**
Officially full time or part time	Full time	Part time	Part time	Full time	Part time	Full time	Part time	Full time	Full time	Part time	Full time	Part time
Employed or volunteer	Employed	Volunteer	Volunteer	Employed	Volunteer	Employed	Volunteer	Employed	Employed	Volunteer	Employed	Volunteer
Length of pre-service training and any degree requirements	9th grade certificate or equivalent and paramedical training	∼10 days, depending on partner priorities and disease burdens	19 days	2 year nursing degree + 2 weeks training	∼5 days	12 weeks	Varying lengths depending on partner support	22 days	6 months	2 weeks	1 year	Varying lengths depending on partner support
Financial compensation												
Salary,^a^ US$	∼300/month			∼130-160/month		∼175 – 280/month		∼ 70/month	∼80/month		∼380/month	
Stipend,^a^ US$		∼75/month (performance based)	∼30/month							∼30/month		<20 hours per week ∼30/month
Incentive amounts			Standardized		Various		Standardized			Various		Standardized
Payment mode	Bank account transfer	Mobile money transfer	Mobile money transfer	Bank account transfer	Cash	Bank account transfer	Cash	Various partner supported modes	Various partner supported modes	Mobile money transfer	Bank account transfer	Cash and Mobile credit
Government supported	X	X	X	X		X			X		X	
Donor supported		X	X	X	X	X	X	X	X	X	X	X
Minimum wage alignment	X	X		X		X		X	X		X	
Functions and services												
Community mobilization	X	X	X	X	X	X	X	X	X	X	X	X
Health promotion	X	X	X	X	X	X	X	X	X	X	X	X
iCCM services	X	Referral only	If >5 km from health facility	Facility-based IMNCI	Referral only	X	Referral only	X		If >5 km from health facility		X
Other services	X	One Health Package	Ad hoc campaigns	X	Ad hoc campaigns	X	Ad hoc campaigns	X	X	X	X	Ad hoc campaigns
Catchment area												
CHW to population ratio	At least 1/arrondissement	1/150–200 households	1/1,000 people	1/5,000 people	Undefined	1/1,000 people	Undefined	1/100–700 people	1/10,000 people	1/5,000 people	1/health post	1/250–500 households
Individual community		X	X		X		X	Based on population density		X		X
Multiple communities	X			X		X		Based on population density	X		X	
Health facility relationship												
Facility based	X			X		X			X		X	
Supervised by health facility staff	X	X	X	X	X	X	X	X	X	X	X	X

Abbreviations: CHW, community health worker; iCCM, integrated community case management; IMNCI, Integrated Management of Newborn and Child Illnesses.

a Equivalent amounts calculated as of June 2023.

### Data Collection

Semistructured interview guides were developed covering the following topics: current policies, mechanisms and variation in CHW compensation (both financial and nonfinancial elements) within the key informant’s country; challenges and lessons learned in designing and implementing CHW compensation schemes; strategies employed by the key informant or others within the country to overcome challenges; and perspectives on which compensation schemes have worked and why, including any perceived relationship to CHW performance (Supplement 1). The semistructured guides for interviews with CHWs differed from the other key informant categories and covered the following topics: description of their CHW role, including time spent on iCCM activities; engagement with communities, health facilities, and the MOH more broadly, including any support received (both financial or nonfinancial); comparison of entitlement versus receipt of financial and nonfinancial compensation, including equipment, supplies, and any in-kind benefits (i.e., how much, how often, from whom); and perceived value of the various forms of financial and nonfinancial compensation received (Supplement 1). The study protocol and data collection tools were developed in English and then translated into French for relevant study countries. Semistructured interview guides were also translated into relevant national or local languages in countries where subnational-level key informant interviews were conducted (i.e., Bambara in Mali; Hausa and Zarma in Niger; Chichewa in Malawi).

It must be emphasized that as a qualitative study with a research aim of understanding the perspectives of a range of stakeholders, interviews were extremely versatile and driven by the expertise of the key informants, not the semistructured guide that was originally prepared. This included focusing on the considerations key informants felt were most relevant to understanding CHW compensation schemes and their effectiveness within their country context, including adapting to the language and concepts they use to describe this. Overall, this approach raised considerations not originally identified when the semistructured guides were developed, which shaped the focus of interviews as concepts were progressively explored with key informants.

All national-level key informant interviews were conducted remotely over Zoom in English or French by 2 members of the research team from March through August 2022. All subnational interviews were conducted in person in Bambara, Chichewa, Hausa, or Zarma by 3 locally recruited research consultants (1 per country) from June through July 2022. All interviews were recorded with the permission of the key informants and then transcribed by the researchers who conducted the interviews. Amberscript software was used for transcription of the national-level interviews recorded in English or French. All subnational interviews were translated into English or French by the locally recruited research consultant and then were reviewed for accuracy by the research team’s respective country focal point.

### Data Analysis

Transcriptions of all interviews were uploaded to Dedoose software for thematic analysis. The research team developed a codebook of deductive codes based on the study objectives and lines of inquiry of the study, then coded all interview transcripts (Supplement 2). Based on the review of coded data, themes were identified across data from each country and then compared for similarities and differences across the 7 countries. Relevant country documentation (i.e., national health policies and strategies related to CHWs and iCCM services, including any documents referenced by key informants in interviews) was reviewed to provide contextual background for the interpretation of qualitative interview data.

### Ethical Approval

Ethics review and approval were obtained from the respective ethics review bodies in each country as follows: Comité National pour l’Éthique de la Recherche en Santé; Ministère de la Santé, Benin (referred this study to Population Service International’s Research Ethics Board determination); Comité d’Éthique pour la Recherche en Santé et Ministère de la Santé, de l’Hygiène Publique et du Bien-Être, Burkina Faso (N°2022-03-043); Ghana Health Service Ethics Review Committee (GHS-ERC 008/03/22); Comité National d’Éthique pour la Santé et les Sciences de la Vie, Ministère de la Santé et du Développement Social, Mali (N°2022076/MSDS-CNESS); National d’éthique pour la Recherche en Santé, Ministère de la Santé Publique, de la Population et des Affaires Sociales, Niger (28/2022/CNERS 16 June 2022); National Health Sciences Research Committee, MOH, Malawi (#22/03/2879); Excellence in Research and Ethics and Science Institutional Review Board, MOH, Zambia (Ref. No. 2022-May-010). The Population Service International’s Research Ethics Board gave the study a determination of “Non-Human Subjects Research” (#18.2022).

## RESULTS

### Overview of Financial Compensation Schemes

The financial compensation schemes currently implemented in each of the 7 countries are summarized in terms of amount, frequency, mode, and type of payment provided to a CHW (Table 2). Each study country used a different health service delivery model that engaged CHWs with a differing mix of responsibilities in terms of service package, catchment area, and health facility relationship. These differences are important for considering how financial compensation relates to the “job demands, complexity, number of hours, training, and roles” that a CHW undertakes (as recommended in the 2018 WHO guideline) and understanding patterns in compensation schemes across study countries.

The majority of the study countries (i.e., Benin, Ghana, Malawi, Niger, and Zambia) employed a dual-cadre service delivery model with a combination of CHWs who were at least partially facility based (i.e., responsible for some activities or services within a health facility, usually serving multiple communities within a catchment area) and other CHWs who were entirely community based (i.e., only responsible for activities or services outside the walls of a health facility, usually 1 community where they also live). Allocation of iCCM service delivery responsibility varied across these dual-cadre service delivery models. The part-time volunteer CHWs in Niger and Zambia were responsible for providing iCCM services, while the part-time volunteer CHWs in Benin, Ghana, and Malawi were not responsible for providing iCCM services and were instead intended to refer children to the full-time employed CHWs for these services. In these 5 countries, CHWs that were at least partially facility based were considered full-time, employed workers and paid through government systems (e.g., civil servant payroll mechanisms). These CHWs received financial compensation as a salary that was paid through a bank account transfer. The CHWs who were entirely community based were considered part-time volunteers and supported primarily through implementing partner systems (i.e., external donor finances and implementing partner arrangements for financial compensation). These CHWs received financial compensation in the form of a stipend or activity-based incentives of varying amounts paid either in cash or mobile phone transfers. In most cases, compensation to CHWs with a volunteer status did not meet levels outlined in minimum wage laws, and these laws were not viewed as legally applicable to them as volunteers.

Benin, Ghana, Malawi, Niger, and Zambia employed a dual-cadre service delivery model with a combination of CHWs who were at least partially facility based and other CHWs who were entirely community based.

Burkina Faso and Mali did not have a dual-cadre CHW model and differed in their financial compensation schemes from patterns observed in the other 5 study countries. In Burkina Faso, entirely community-based CHWs (i.e., agents de santé à base communautaire) provided iCCM services (if based in a community at least 5 km from a health facility) and were considered part-time volunteers. However, the government set a standard financial compensation rate for all agents de santé à base communautaire and contributed 75% of this cost from the government budget, with the other 25% coming from external donors. In Mali, under the country’s community health strategy (2021–2025), entirely community-based CHWs (i.e., agents de santé communautaire) who provided iCCM services were considered full-time employees and were specified to receive a monthly salary greater than or equal to the Guaranteed Interprofessional Minimum Wage (approximately US$70). However, domestic public financing is yet to be secured for this purpose; thus, all financing came from external donors. Mali previously employed a dual-cadre model, but the community-based, part-time, volunteer CHWs (i.e., relais communautaire) were being phased out under the 2021–2025 strategy.

### Effectiveness of Financial Compensation Schemes

Key informants described the effectiveness of financial compensation schemes primarily in terms of 3 considerations: (1) regularity of payment, how routinely CHWs received various kinds of compensation; (2) consistency of distribution, how uniformly it was provided across individuals of a particular type of CHW; and (3) sufficiency of amount, how adequate or fair the compensation amount was deemed to be by the key informant. These 3 considerations were described from key informants’ perspectives as ultimately impacting health worker performance and service delivery.

Key informants did not raise concerns about the effectiveness of financial compensation schemes when CHWs were considered employees and paid salaries through bank account transfers. Regularity of payment and consistency of distribution were considered essentially a given in these cases. This was stated explicitly by multiple key informants in Ghana and Malawi in reference to payment of civil servant community health officers and health surveillance assistants (i.e., the full-time employed CHWs responsible for providing iCCM services in Ghana and Malawi, respectively). Additionally, sufficiency of amount was rarely raised as an issue by key informants in these cases, although adequate equipment, supplies, and transport were still noted as challenges for CHW performance and service delivery. Several key informants also raised the need for other employment benefits, such as housing, health insurance, and retirement pensions.

Key informants raised concerns about the effectiveness of financial compensation schemes when CHWs were considered volunteers and received various forms of financial and nonfinancial incentives, which were noted to involve a range of challenges. In these cases, regularity of payment was often described as dependent on arrangements with individual donors or implementing partners, which resulted in gaps in payment between the beginning and end of projects. This situation was also noted to cause a sense of anxiety and uncertainty, especially for MOH health service administrators and health facility managers, who depended on CHWs to continue provision of health services within their catchment areas. These arrangements were also noted to impact the consistency of distribution, with some CHWs receiving certain stipends or activity-based incentives while other CHWs did not. The sufficiency of incentive amounts was frequently raised as a concern in these cases as well. Overall, these elements of irregularity, inconsistency, and insufficiency in compensation were noted to demotivate CHWs and cause attrition over time, which, in turn, negatively affected the continuity and quality of CHW services, particularly the provision of iCCM services.

Key informants raised concerns about the effectiveness of financial compensation schemes when CHWs were considered volunteers and received various forms of financial and nonfinancial incentives.

*What would be a fair way to compensate people who are working, even though we call them volunteers? I mean, they should be compensated if they’re doing work within the community, which benefits the communities and the government.* —Donor informant, Zambia

*The 7,500 CFA franc [incentive amount received] is not enough because the activity of the iCCM does not allow us to take care of ourselves and our families. We would like our living conditions to be improved in order to continue this voluntary work for which we have willingly committed ourselves.* —CHW informant, Niger

*In fact, the payment of incentives to the [CHW] allows them to be loyal and to ensure the sustainability of the interventions…It prevents them from being tempted to go outside, to give up and so on. It allows us to guarantee the quality of care, the level of services.* — Government informant, Niger

Overall, key informants across all informant categories and study countries commonly raised the need for CHWs, including those functioning as volunteers, to have a livelihood, provide for their families, and be fairly compensated for their work. Key informants did not suggest specific compensation amounts that they would consider sufficient. However, key informants in some countries, particularly Benin, Mali, and Niger, expressed the view that CHW compensation should align with the guaranteed minimum wage law or guaranteed minimal interprofessional salary scales. These rationales were used by key informants to describe whether a particular compensation amount was deemed sufficient.

Key informants described a variety of initiatives across study countries specifically to address concerns around the effectiveness of compensation schemes for volunteer status CHWs, including the following: establishment of pooled funds for volunteer compensation with contributions from both government and donors in Benin and Niger; development of volunteer contracts and incentive guidelines for all implementing partners in Zambia; nationwide government managed mobile payment mechanisms in Benin and Niger; and volunteer stipend standardization with a cost-sharing arrangement between government and donors in Burkina Faso. These initiatives were described primarily as useful for improving the regularity of payments and consistency of distribution, although combining government and donor resources was also described as a means to improve sufficiency of amount.

### Lessons Learned From Countries’ Experiences

Across the 7 study countries, key informants described a variety of lessons learned based on their experiences with designing and implementing CHW financial compensation schemes. Five key themes emerged from their descriptions: the need for government leadership; coordination across programs and ministries; engagement with CHWs, community leaders, and broader communities; harmonization of external donor and partner support; and realistic transitional financing plans.

#### Establish Government Leadership

Key informants noted the need to establish the government’s role and leadership in CHW financial compensation, both in terms of providing policy direction and governance mechanisms in implementation. The concept of leadership was also linked with ownership. Some key informants described dynamics where donors and implementing partners were seen as controlling CHW motivation with their resources and were viewed as the assumed financial support of community health indefinitely, thus stifling government ownership.

*For a long time, it’s been donors and other external organizations that have been supporting community-based work. It may be a perception in government that, okay, this is a donor issue and they’ll continue doing that in perpetuity. But I think that mindset needs to be changed. The government itself needs to realize that these people are actually essential for the implementation of government programs, and they are providing services that benefit the people of Zambia.* —Donor informant, Zambia

#### Promote Coordination Across Programs and Ministries

Key informants noted the need for coordination across national programs and between ministries to secure budget commitments and manage financial compensation systems. One donor key informant, who was formerly in a government role, described how external donors sometimes worked with government officials in ways that did not encourage coordination, which, in turn, limited long-term, institutionalized arrangements.

Key informants noted the need for coordination across national programs and between ministries to secure budget commitments and manage financial compensation systems.

*So […] a donor is working with a particular program and there’s a government public staff member who’s their main counterpart, their main focal point, and then that person ends up making a lot of decisions, maybe a little bit unilaterally. Sometimes when you have one person making a lot of decisions, it affects the other people. And what it means is that immediately when you end your project that person probably doesn’t have that power or the resources that were available to him, so that is the end of your activities too…But of course, when I was part of the Ghana Health Service, I also benefited like that.* —Donor informant, Ghana

#### Engage Community Health Workers, Community Leaders, and Broader Communities

Key informants highlighted how involvement of CHWs, community leaders, and broader communities in financial compensation discussions and planning meetings was valuable. This was noted as important for understanding the needs of CHWs and the priorities of community members. The involvement of community leaders and other community members was noted to help ensure community buy-in from the beginning and avoid misunderstandings about the CHW’s role and their compensation. Community leaders were also noted to be influential in advocating for allocation of resources to support CHWs in their communities.

*We need to involve the community-based volunteer. I think that is one of the best practices. Their involvement at the planning level is quite, quite key*. —Government informant, Zambia

#### Harmonize External Donor and Partner Support

Key informants repeatedly expressed the need to harmonize donor and partner approaches to CHW financial compensation. Several key informants described using committees and working groups comprised of government and partner representatives to develop strategies and align efforts for a harmonized approach to CHW financial compensation. Challenges were noted both in terms of partner alignment on compensation amounts and support for nationwide mechanisms managed by the government (e.g., volunteer incentive guidelines, mobile payment systems, pooled funds).

*I think that the most important thing is really the alignment, the harmonization of all the partners with what is defined as strategies or as an operational plan for the country. That is really very important to also define a payment mechanism that could be applied to all partners.* —UN informant, Niger

*A guidance document [is important]…to say that, instead of dispersing the efforts we try to put the efforts together and go in the same direction while staying in our objective to ensure the activities of [the national community health service package] and incentives.* —Government informant, Mali

#### Develop Realistic and Transitional Financing Plans

Key informants emphasized the need for government leadership in designing and implementing CHW compensation schemes, including approaches for financing. Several key informants also emphasized the need for realistic domestic financing goals, which could be supported by government and donors working together in the development of transitional financing plans for CHW compensation. Developing realistic transitional financing plans was considered important for all types of CHW compensation schemes, including those involving employed or volunteer status CHWs.

*It’s true that the ideal would have been the state budget alone, but we know the context of our country. We need support. This may be the long-term challenge of partner support. It will be necessary that perhaps the country also tries to see progressively, to see perhaps how to reduce this contributory share to be able to really establish something perennial.* —Government informant, Burkina Faso

*A large number of those community health assistants [full-time, employed CHWs in Zambia] ended up not being employed or losing their jobs essentially. So that is a lesson learned. I mean, if we are looking at sustainability and transitioning. Transitioning support for community-based workers to the government. We should bear in mind that there may be challenges with the government actually taking them on. We need to actually engage the government on how we can develop a transition plan for the staff that we are supporting and how it can be done in a phased manner, which will allow the government to mobilize resources in order to take on the staff that would be transitioning to them*. —Donor informant, Zambia

## DISCUSSION

This study found that CHW compensation schemes associated with an employed worker status and government payroll mechanisms were most often perceived as effective by key stakeholders, while other types of compensation schemes associated with a volunteer status varied widely in their delivery mechanisms (e.g., cash or mobile phone distribution) and were perceived as less effective. These findings are consistent with a recent systematic review of dual-cadre CHW programs (i.e., those with both salaried and unsalaried CHWs), which found that unsalaried CHWs frequently reported nonpayment, inadequate or inconsistent payment of incentives, and an overburdensome workload.^7^

Minimum wage laws and other legal protections for workers are often not applied to CHWs labeled as volunteers.^8^ However, the International Labor Organization has called for the recognition, reduction, and redistribution of unpaid care work as well as the promotion of decent working conditions and representation for all care workers.^9^ There is growing evidence in favor of a shift toward the professionalization of CHWs for improved health system outcomes. For example, a study conducted by Ballard et al. on the continuity of community-based healthcare provision during the COVID-19 pandemic concluded that CHWs who received compensation packages in line with WHO recommendations (including financial remuneration, training, supplies, and appropriate supervision) were able to maintain the same level of service provision as in nonpandemic times; thus, providing this kind of support to professionalized CHWs may contribute to better pandemic preparedness in the future.^10^

This study included countries with varying CHW arrangements, including those with CHWs recognized as employed workers and those recognized as volunteers. Regardless of the recognized status of the CHWs, key informants used the same considerations (i.e., regularity of payment, consistency of distribution, and sufficiency of amount) for describing the effectiveness of CHW compensation schemes in terms of impact on health worker performance and service delivery continuity. It is notable that key informants did not raise concerns about the effectiveness of financial compensation schemes when CHWs were considered employees but did raise concerns when CHWs were considered volunteers. Compensation delivery mechanisms used with CHWs considered to be employed workers were largely the same across countries (i.e., bank account transfers, government payroll systems), while delivery mechanisms varied substantially for CHWs considered to be volunteers (i.e., a combination of cash-based or mobile facilitated payment systems often dependent on implementing partners). Efforts to overcome problems with regularity of payment, consistency of distribution, and sufficiency of amount for CHWs recognized as volunteers varied across countries, but all involved greater involvement of government in management and financial investment (e.g., establishment of pooled funds and cost-sharing arrangements between donors and government, government-led standardization of incentive or stipend amounts, nationwide government managed mobile payment mechanisms).

Efforts to overcome problems with regularity of payment, consistency of distribution, and sufficiency of amount for CHWs recognized as volunteers varied across countries.

This study was designed to focus on financial compensation schemes implemented for CHWs providing iCCM services, so the findings of this study are most applicable to CHWs engaged in routine, continuous health service delivery (e.g., iCCM services) rather than those only engaged in ad-hoc or seasonal activities (e.g., occasional health campaigns or community mobilization events). At the time interviews with key informants were conducted, half of the part-time, volunteer-status CHWs across countries in this study were responsible for the routine task of iCCM service delivery (i.e., agents de santé à base communautaire in Burkina Faso; relais communautaire in Niger; community-based volunteers in Zambia).^11^^–^^13^ Thus, key informants described their perceptions of the effectiveness of compensation schemes for part-time volunteer CHWs with the need to deliver routine iCCM services in mind. Additionally, Benin had recently shifted the responsibility of iCCM services to a new full-time, employed CHW (i.e., agents de santé communautaire qualifié), and Mali had just moved to recognize CHWs responsible for providing iCCM services (i.e., agents de santé communautaire) as full-time, employed health workers.^14^^,^^15^ Key informants in both Benin and Mali consistently expressed support for these policy changes. Meanwhile, Ghana and Malawi have long allocated iCCM service delivery responsibilities to full-time, employed CHWs.^16^^,^^17^ Policymakers across countries in this study appear to be increasingly shifting responsibility for the provision of iCCM services to full-time, employed CHWs with salaries and government payroll mechanisms.

None of the key informants interviewed referenced the 2018 WHO recommendation on CHW remuneration nor explicitly described the factors the recommendation suggests for determining “commensurate” remuneration (i.e., job demands, complexity, number of hours, training, and roles) within their own descriptions of their country’s CHW compensation schemes or effectiveness of those schemes. Some key informants generally described the high levels of responsibility and time commitment for CHWs, especially when needing to be continuously available to provide iCCM services when a child was ill. However, beyond the official recognition of a CHW role being full-time or part-time, most key informants were not able to confidently or concretely describe CHW time commitments, particularly those in roles labeled as “part-time” or “volunteer.” Additionally, estimated time commitments described by CHW key informants varied and were difficult to quantify through key informant interviews. The extent of training or complexity of tasks were not raised as a consideration by key informants, although some praised CHWs for the difficult and important work that they do. Greater sensitization of the 2018 WHO recommendation on CHW compensation, along with practical guidance on how to assess CHW “job demands, complexity, number of hours, training, and roles,” may be needed to support the practical application of this global normative guidance at the country level.

### Limitations

Resource constraints made it necessary to limit the total number of key informants who could be interviewed, so it was only possible to conduct subnational interviews (i.e., interviews with subnational MOH administrators, health facility staff, and CHWs) in 3 of the 7 study countries. Additionally, resource constraints made it necessary to conduct national interviews remotely (i.e., via Zoom or phone). Conducting interviews remotely presented challenges, including lack of visual cues and connectivity problems, which likely impacted the quality of interviews to some extent. In a few cases, numerous attempts to connect by Zoom or phone over a period of multiple weeks were not successful, so interviews could not be conducted with the potential key informant. These constraints resulted in significant variation in the total number of key informant interviews per country. However, interviews also varied significantly in length and qualitative richness (i.e., depth and detail of information provided by the key informant). This meant that more information and insight were gained from a fewer number of interviews in some cases (e.g., this was particularly true in Ghana, where the fewest number of key informant interviews per country took place, but those conducted were particularly lengthy and qualitatively rich).

The findings of this study are based on patterns identified through thematic analysis of transcripts from interviews with a total of 68 key informants. Key informant selection prioritized government policymakers, national program managers, and others directly responsible for iCCM and other community-based health services, particularly those with extensive, long-term experience working within their country’s health system. This prioritization resulted in fewer key informants from donor, NGO, or UN categories. Therefore, the study’s findings on perceptions of effectiveness are most reflective of individuals with the key informant background prioritized for this study.

Overall, the total number of interviews was sufficient to reach a point of qualitative data saturation for the main lines of inquiry explored in this study, namely documentation of CHW compensation schemes, perceived effectiveness, challenges, and lessons learned. However, the study sample did not yield significant granular distinctions in patterns between categories of key informants (e.g., civil society, donor, or NGO representatives; government administrators, service providers, and policymakers; different types of CHWs). At the end of each interview, key informants were asked to suggest names of other stakeholders working in the community health space for further interviews; this referral-based recruitment method may have introduced selection bias. For further analysis, we recommend conducting more key informant interviews at a subnational level across all countries in the study, including with a broader range of CHWs.

## CONCLUSION

This study documented the financial compensation schemes implemented for CHWs providing iCCM services across 7 countries in sub-Saharan Africa and found that key informants perceived compensation schemes as effective when payment is regular, distribution is consistent, and the amount is sufficient to support health worker performance and service delivery continuity. CHW compensation schemes associated with an employed worker status and government payroll mechanisms are most often perceived as effective by key informants, while other types of compensation schemes associated with a volunteer CHW status vary widely in their delivery mechanisms and are perceived as less effective. Policymakers should consider these findings in the design of compensation schemes for CHWs engaged in routine, continuous health service delivery (e.g., iCCM services) within the context of their country’s health service delivery model. Systematic documentation of the tasks and time commitment of CHWs, particularly those with a volunteer status, could support more recognition of their health system contributions and better determination of commensurate compensation in line with the 2018 WHO recommendation.

## Supplementary Material

GHSP-D-24-00008_Supplements.pdf

## Notes

**Peer Reviewed**
