# Supplementary material for: Documenting Community Health Worker Compensation Schemes and Their Perceived Effectiveness in Seven sub-Saharan African Countries: A Qualitative Study
Source: Glob Health Sci Pract. 2024 Jun 27;12(3):e2400008. doi: 10.9745/GHSP-D-24-00008 (PMC11216702; doi:10.9745/GHSP-D-24-00008)
Supplement: GHSP-D-24-00008_Supplements.pdf [file GHSP-D-24-00008_Supplements.pdf]

Supplement to: Davis AL, Flomen L, Ahmed J, et al. Documenting community health worker compensation schemes and their perceived effectiveness in seven sub-Saharan African countries: a qualitative study. Glob Health Sci Pract. 2024;12(3):e2400008. https://doi.org/10.9745/GHSP-D-24-00008

Supplement 1. Semistructured Guides for Key Informant Interviews

Note: *Questionnaires were developed based on a broad literature review and understanding of the questions that might be relevant to explore the study's lines of inquiry with key informants. As specified in the study protocol, these questionnaires were intended to be used as semistructured interview guides with the flexibility to respond to the background, knowledge, experience and perspectives of key informants as the research team sought to build a meaningful, qualitative understanding of stakeholder's views on CHW compensation schemes, perceived effectiveness, challenges and lessons learned.*

Donor Field level Questionnaire (e.g., UNICEF, GF, GFF, EU, WHO, USAID, PMI)

Donor Field Interview Guide // CHW Financial Compensation Schemes -- Assessment

Interview date

**Interview date**	
**Respondent name**	
**Position**	
**Organization**	
**Respondent category**	
**Additional information about the respondent**	
**Interviewer name**	
**Start time**	
**End time**	

Notes for Interviewer:

- *Before asking the questions below, provide background on the assessment and go through the informed consent process. If the respondent has already signed and returned a completed consent form, provide a quick overview of the purpose of the assessment and the consent form.*
- *Ask if you have consent for the interview to be recorded If not, ensure you have good notes].*
- *When you have reached 45 minutes, inform the respondent about how many questions remain and check if you can complete the questions within the 15 minutes, or ask for additional time if necessary.*
- *Listen closely to the respondent's answers and only ask those questions that are relevant to their role and function (e.g., if the respondent doesn't engage in capacity building, those questions may not be relevant)*

BEGIN RECORDING

Introduction

1. I'd like to start by hearing a bit about your own and your organization's work in community health and iCCM, particularly related to financial compensation. Can you tell me a bit about your program(s), your role, and how you are engaged in iCCM or with CHW financial compensation in the country you work in?
   Probe for: technical and financial assistance, and the kinds of financial assistance

2. Does your organization's work in community health include compensation for CHWs?
   If YES:
   a. What prompted your organization to engage in CHW compensation?

3. How long has your organization been involved in giving compensation for CHWs in the [COUNTRY] you work in?

4. What are the types of compensation provided by your organization?
   Probe for: in kind, financial or other incentives or compensation

5. Please explain how CHW financial and non-financial compensation activities are supported by your organization work.
   Probe for: payments directly to CHW? To local government? Through the Ministry (and which one – Health, Finance, other)? Through a local or international NGO? Pooled funds for the compensation in one basket?

6. Do you feel compensation has improved CHW performance and/or decreased attrition?
   Probe for: what evidence do they have; ask for reports or studies that document improvement and/or reduced attrition.

7. What roadblocks, challenges or barriers have you encountered related to design and implementation of compensation schemes for ICCM?

8. What do you consider to be the key lessons learned and best practices over time from your organization's experience with CHW compensation?

9. How would you define sustainability in terms of iCCM financial compensation schemes?

10. What mechanisms or strategies is your organization supporting to a sustainable path for compensation?

11. What impact has the Covid-19 pandemic had on your organization policy for compensation for CHW working on ICCM/CCM?
    Probe for: reduction of compensation due to the Covid-19 pandemic

12. What do you see as the future of Community health, namely ICCM/CCM and the CHW in [COUNTRY] you work in?

13. Is there anything else you feel would be important for me to know?

14. Are there any documents we should include in our review?

15. What questions do you have for me?

Thank you for your time.

Supplement to: Davis AL, Flomen L, Ahmed J, et al. Documenting community health worker compensation schemes and their perceived effectiveness in seven sub-Saharan African countries: a qualitative study. Glob Health Sci Pract. 2024;12(3):e2400008. https://doi.org/10.9745/GHSP-D-24-00008

National level Country Question [MOH, Ministry of Finance, other relevant ministries)

Ministry of Health Interview Guide // CHW Financial Compensation Schemes -- Assessment

**Interview date**	
**Respondent name**	
**Position**	
**Organization**	
**Respondent category**	
**Additional information about the respondent**	
**Interviewer name**	
**Start time**	
**End time**	

Notes for Interviewer:

- *Before asking the questions below, provide background on the assessment and go through the informed consent process. If the respondent has already signed and returned a completed consent form, provide a quick overview of the purpose of the assessment and the consent form.*
- *Ask if you have consent for the interview to be recorded. If not, ensure you have good notes]*
- *When you have reached 45 minutes, inform the respondent about how many questions remain and check if you can complete the questions within the 15 minutes, or ask for additional time if necessary.*
- *Listen closely to the respondent's answers and only ask those questions that are relevant to their role and function (e.g., if the respondent doesn't engage in capacity building, those questions may not be relevant)*

BEGIN RECORDING

Introduction

1. I'd like to start by hearing a bit about your work with [*your unit*]. Can you tell me a bit about your program(s), your role, and how you are engaged in iCCM or in CHW compensation efforts in [your country]
   Probe for: confirmation of cadre of worker designated to carry out iCCM in general and for malaria and which department(s) coordinate(s)s iCCM?

2. Could you please describe the policy on how CHWs are compensated in general and for iCCM?
   Probe for: Is this part of a CHW policy or a broader policy? What types of financial and non-financial compensation is the health system responsible for providing? Does this financial compensation include iCCM? Is the iCCM compensation policy in line with country minimum wage policies?

3. Please describe the role of community engagement in CHW financial and non-financial compensation.

   Probe for: If there is a community engagement, can you give me a few concrete examples of the role of community engagement in CHW compensation, such as non-financial, in-kind, or financial?

	**From whom? (HS/Community/-Other)**		
		**In the policy?**	**Being implemented**
**Financial**			
Cash incentive for services rendered			
Direct and regular salary payment			
Allowance/benefit for transport and trainings			
Performance-based financial rewards			
Access to microcredit			
Others (specify)			
**Non-financial**			
Trust in the community			
Is part of local councils			
Others (specify)			
**In-kind**			
Special privileges, such as access to free social services			
Goods, animals, food, gifts, etc			
Equipment: bicycles, umbrella, badges, uniforms, mobile phones, etc			
Others (specify)			

4. What roadblocks, challenges or barriers did you encounter in **designing** financial compensation for i CCM? [FOR THOSE NOT INVOLVED DIRECTLY IN DESIGN] what challenges/barriers are you aware of related to designing the compensation policy, systems and processes?

   Probe for: How do you establish who gets compensated?

5. [IF THE COUNTRY ALSO HAS NON-FINANCIAL COMPENSATION, ASK..] What roadblocks, challenges or barriers did you encounter in **designing** non-financial compensation for iCCM?

6. What kind of roadblock, challenges or barriers have you encountered in **implementing** the financial compensation policy?

   Probe for: information systems that track the number and location of CHWs
   Probe for: Specify roadblock specifically for both financial and non-financial compensation
   Probe for: At which level did these roadblocks occur– national, regional, district, local level (health center), community?
   Probe for: Any issues with regularity and consistency of pay outs of compensation
   Probe for: Any ways in which implementation differed from the design

7. What roles do national, subnational and community actors play in implementing the financial compensation policy?

    Probe for: Who is responsible for each level to distribute the iCCM compensation?
    Probe for: Are the different levels in contact with each other to facilitate the distribution?
    Probe for: What kind of mechanisms do they use to coordinate and communicate about the compensation?

8. Of the various issues/barriers/roadblocks, which ones would you say are the most important and why?

    Probe for: Those that are the most important to the least important and why

9. What impact has the Covid-19 pandemic had on the work that the CHWs do on iCCM? What impact has it had on the financial compensation distribution?

    Probe for: What kinds of challenges or adaptations did you see in iCCM implementation during the pandemic?
    Probe for: Were there specific impacts on compensation? Were they still given? Reduced?

10. Thinking about the obstacles (roadblocks, challenges, barriers) we just discussed, what strategies did [COUNTRY] use to address these?

    Probe for: What strategies (if there are) were developed for each roadblock (even if they overlap with each other)

    Probe for: How effective was each strategy? How did you ascertain its effectiveness?

11. What aspects of the CHW financial and non-financial compensation in the context of iCCM do you feel have worked best?

12. What lessons can be derived from [COUNTY]'s experience in implementing financial and non-financial compensation for iCCM/CCM?

    Probe for: those that are relevant specifically for [COUNTRY]
    Probe for: For each one, how have they informed the implementation or the policy?

13. How would you define sustainability in terms of iCCM financial compensation?

14. To what degree do you feel these iCCM financial compensation are sustainable?

    Probe for: What feels sustainable and why?
    Probe for: What concerns do you have about sustainability?

15. How relevant do you think [COUNTRY]'s best practices related to iCCM financial and non-financial compensation are for other countries?
    Probe for: Why or why not?

Probe for: both financial and non-financial compensation

16. Are there aspects of your financial compensation scheme that must stay the same to be feasible/sustainable in another country context?
    Probe for: What can be modified without losing integrity of the strategy

17. How do the iCCM financial compensation vary from those in other health sector activities?
    Probe for: Could the strategies and lessons learned be applied to community-based activities?

18. Is there anything else you feel would be important for me to know?

19. Are there any documents we should include in our review?

20. What questions do you have for me?

Thank you for your time.

Supplement to: Davis AL, Flomen L, Ahmed J, et al. Documenting community health worker compensation schemes and their perceived effectiveness in seven sub-Saharan African countries: a qualitative study. Glob Health Sci Pract. 2024;12(3):e2400008. https://doi.org/10.9745/GHSP-D-24-00008

Subnational level Country Questionnaire [Regional and District Actors supporting iCCM CHW financial compensation]

Subnational level Interview Guide // CHW Financial Compensation Schemes – Assessment

**Interview date**	
**Respondent name**	
**Position**	
**Organization**	
**Respondent category**	
**Additional information about the respondent**	
**Interviewer name**	
**Start time**	
**End time**	

Notes for Interviewer:

- *Before asking the questions below, provide background on the assessment and go through the informed consent process. If the respondent has already signed and returned a completed consent form, provide a quick overview of the purpose of the assessment and the consent form.*
- *Ask if you have consent for the interview to be recorded. If not, ensure you have good notes]*
- *When you have reached 45 minutes, inform the respondent about how many questions remain and check if you can complete the questions within the 15 minutes, or ask for additional time if necessary.*
- *Listen closely to the respondent's answers and only ask those questions that are relevant to their role and function (e.g., if the respondent doesn't engage in capacity building, those questions may not be relevant)*

BEGIN RECORDING

Introduction

1. I'd like to start by hearing a bit about your work with [*your unit*]. Can you tell me a bit about your program(s), your role, and how you are engaged in iCCM or in CHW financial and non-financial compensation efforts in [your country]
   Probe for: confirmation of cadre of worker designated to carry out iCCM in general and for malaria and which department(s) coordinate(s)s iCCM?

2. Can you please describe the policy on CHW financial compensation, particularly for iCCM?
   a. *Probe for:* Is this part of a CHW policy or a broader policy?
   b. *Probe for:* What types of compensation is the health system responsible for providing? Do these compensations include iCCM?

3. Please describe the role of community engagement in CHW financial and non-financial compensation.
    a. *Probe for:* If there is a community engagement, can you give me a few concrete examples of the role of community engagement in CHW compensation, such as non-financial, in-kind, or financial?
    b. *Probe for:* If the concrete example is given, how do you make sure that these examples, compensation are really executed?

	**From whom? (HS/Community/-Other)**		
		**In the policy?**	**Being implemented**
**Financial**			
Cash incentive for services rendered			
Direct and regular salary payment			
Allowance/benefit for transport and trainings			
Performance-based financial rewards			
Access to microcredit			
Others (specify)			
**Non-financial**			
Trust in the community			
Is part of local councils			
Others (specify)			
**In-kind**			
Special privileges, such as access to free social services			
Goods, animals, food, gifts, etc			
Equipment: bicycles, umbrella, badges, uniforms, mobile phones, etc			
Others (specify)			

4. What roadblocks, challenges or barriers did you encounter in implementing financial and non-financial compensation for ICCM, as stated in the policy at your level? what challenges/barriers are you aware of related to implementing financial and non-financial compensation policy, systems and processes?
    a. *Probe for:* How do you establish who gets compensated?

5. What kind of roadblock, challenges have you encountered in implementing the financial compensation policy?
    a. *Probe for:* Specify roadblock specifically for both financial and non-financial compensation
    b. *Probe for:* At which level did these roadblocks occur– national, regional, district, local level (health center), community?
    c. *Probe for:* Any issues with regularity and consistency of pay outs of financial compensation

6. What responsibilities and roles does your level play in implementing the financial compensation policy?
    a. *Probe for:* Who is responsible at your level to distribute the iCCM compensation?
    b. *Probe for: Is your level in contact with the level above and below you to facilitate the distribution?*
    c. *Probe for:* What kind of mechanisms do they use to coordinate and communicate about the compensation?

7. Of the various issues/barriers/roadblocks, which ones would you say are the most important?
    a. Probe for: Those that are the most important to the least important and why

8. What impact has the Covid-19 pandemic had on the work that the CHWs do on iCCM ? What impact has it had on distribution of financial compensation?
    a. Probe for: What kinds of challenges or adaptations did you see in iCCM implementation during the pandemic?
    b. Probe for: Were there specific impacts on compensation? Were they still given? Reduced?

9. Thinking about the obstacles (roadblocks, challenges, barriers) we just discussed, what strategies did [COUNTRY] use to address these?
    a. *Probe for:* What strategies (if there are) were developed for each roadblock (even if they overlap with each other)
    b. *Probe for*: How effective was each strategy? How did you ascertain its effectiveness?

10. What aspects of the CHW financial and non-financial compensation in the context of iCCM do you feel have worked best?

11. What lessons can be derived from [COUNTRY]'s experience in implementing financial compensation for iCCM/CCM?
    a. *Probe for:* those that are relevant specifically for [COUNTRY]
    b. *Probe for*: For each one, how have they informed the implementation or the policy?

12. How would you define sustainability in terms of iCCM financial and non-financial compensation?

13. To what degree do you feel these iCCM financial are sustainable?
    a. *Probe for:* What feels sustainable and why?
    b. *Probe for:* What concerns do you have about sustainability?

14. How do the iCCM financial compensation vary from those in other health sector activities?
    a. *Probe for: Are you aware of other compensation in other health sectors at your level?* Could these strategies and lessons learned be applied to community-based activities?

15. Is there anything else you feel would be important for me to know?

16. Are there any documents we should include in our review?

17. What questions do you have for me?

Thank you for your time.

CHW Questionnaire

CHW level Interview Guide // CHW Financial Compensation Schemes – Assessment

**Interview date**	
**Respondent name**	
**Position**	
**Organization/ District/Health Center**	
**Respondent category**	
**Additional information about the respondent**	
**Interviewer name**	
**Start time**	
**End time**	

Notes for Interviewer:

- *Before asking the questions below, provide background on the assessment and go through the informed consent process. If the respondent has already signed and returned a completed consent form, provide a quick overview of the purpose of the assessment and the consent form.*
- *Ask if you have consent for the interview to be recorded. If not, ensure you have good notes]*
- *When you have reached 45 minutes, inform the respondent about how many questions remain and check if you can complete the questions within the 15 minutes, or ask for additional time if necessary.*
- *Listen closely to the respondent's answers and only ask those questions that are relevant to their role and function (e.g., if the respondent doesn't engage in capacity building, those questions may not be relevant)*

BEGIN RECORDING

Introduction

1. I'd like to start by hearing a bit about your work as a CHW. Can you tell me a bit about your role and what you do.
    a. *Probe for:* their role in iCCM, and what other activities they do as a CHW
    b. *Probe for:* How long have you been a CHW/engaged in community health activities?

2. How much time do you spend each week on iCCM activities as a CHW in your community(ies)?

3. Do You ever pay out of pocket for some items either for your own use on the job or for the community/patient's use?
    Probe for: transportation or other out of pocket costs

4. In what ways is the health facility engaged with and supporting you on iCCM?
    a. *Probe for:* Do they come and see you undertaking your task? Provide supplies? Provide medicines? Provide supervision and training?

5. In what ways is the community engaged with and support you on iCCM?
    a. *Probe for:* Do they come and see you undertaking your task? Do they help with other activities? Provide supplies? Provide medicines? Is there a community health committee? What is its role?

6. Do you have a formal agreement with the MOH or the health center or the community?
    a. If yes, is there some written documentation of this agreement? What does the agreement include? SOW? Job description? Level and conditions of financial compensation? Ask to see it

7. Which of these incentives do you currently receive or are supposed to receive [interviewer to list each incentive individually and probe for each column in table]?

    a. *Probe for:* whether this is for them as a CHW in general, specifically for iCCM, different for different programs they support,
  a. *Probe for:* whether the set of incentives has changed over time or the regularity of payments over time

	From whom? (HS/Comm/-Other)	Supposed to receive		Actually receiving		Appreciate incentive (among those receiving)
		How much	How often	How much	How often	
**Financial**						
Cash incentive for services rendered						
Direct and regular salary payment						
Allowance/benefit for transport and trainings						
Performance-based financial rewards						
Access to microcredit						
Others (specify)						
**Non-financial**						
Trust in the community						
Is part of local councils						
Others (specify)						
**In-kind**						
Special privileges, such as access to free social services						
Goods, animals, food, gifts, etc						
Equipment: bicycles, umbrella, badges, uniforms, mobile phones, etc						
Others (specify)						

Among the ones you receive, which ones do you value the most?

8. What impact has the Covid-19 pandemic had on your work for iCCM and the incentives?
   Probe for: What kinds of challenges or adaptations did you see in iCCM implementation during the pandemic?

   Probe for: Were there specific impacts on financial and other incentives/? Were they still given? Reduced? Delayed?

9. Is there anything else you feel would be important for me to know?

10. Are there any documents we should include in our review?

11. What questions do you have for me?

Thank you for your time.

Questionnaire pour les donateurs sur le terrain (par ex. UNICEF, FM, GFF, UE, OMS, USAID, PMI)

Guide d'entretien des donateurs sur le terrain // Système de compensation financière des ASC – Évaluation

Date de l'entretien

**Date de l'entretien**	
**Nom du répondant**	
**Poste**	
**Organisation**	
**Catégorie du répondant**	
**Informations supplémentaires sur le répondant**	
**Nom de l'enquêteur**	
**Heure de début**	
**Heure de fin**	

Notes pour l'enquêteur :

- *Avant de poser les questions ci-dessous, donnez le contexte de l'évaluation et passez en revue le processus de consentement informé. Si le répondant a déjà signé et retourné un formulaire de consentement rempli, fournissez une rapide vue d'ensemble de l'objectif de l'évaluation et du formulaire de consentement.*
- *Demandez si vous avez le consentement pour enregistrer. Dans le cas contraire, veillez à prendre des notes de qualité].*
- *Au bout de 45 minutes, informez le répondant du nombre de questions restantes et voyez si vous pouvez terminer les questions durant les 15 minutes restantes, ou demander plus de temps si nécessaire.*
- *Écoutez attentivement les réponses du répondant et ne posez que les questions pertinentes par rapport à son rôle et sa fonction (par ex., si le répondant n'est pas impliqué dans le développement de capacités, ces questions peuvent ne pas être pertinentes)*

COMMENCEZ À ENREGISTRER

Introduction

1. Pour commencer, j'aimerais que vous me parliez un peu de votre travail et de celui de votre organisation en matière de santé communautaire et de la PCIME-C, en particulier en ce qui concerne la compensation financière. Pouvez-vous me parler un peu de votre/vos programme(s), votre rôle, et de comment vous êtes impliqué dans la PCIME-C ou dans la compensation financière des ASC dans le pays où vous travaillez ?

   Sonder pour : l'assistance technique et financière et les types d'assistance financière

2. Le travail de votre organisation en matière de santé communautaire comprend-il une compensation pour les ASC ?
   Si OUI :
   a. Qu'est-ce qui a incité votre organisation à instaurer une compensation pour les ASC ?

3. Depuis combien de temps votre organisation est-elle impliquée dans l'utilisation d'une compensation pour les ASC dans le [PAYS] où vous travaillez ?

4. Quels types de compensation sont fournies par votre organisation ?
   Sonder pour : en nature, financière, ou autres incitations ou compensations

5. Veuillez expliquer comment les activités de compensation financière et non financière des ASC sont soutenues par le travail de votre organisation. *Sonder pour :* un paiement directement aux ASC ? Au gouvernement local ? Par le biais du ministère (et lequel – santé, finances, autre) ? Par le biais d'une ONG locale ou internationale ? Des fonds communs pour la compensation dans une même enveloppe ?

6. Pensez-vous que la compensation a amélioré les performances et/ou réduit les départs des ASC ? *Sonder pour :* de quelles données factuelles disposent-ils ; demander des rapports ou études qui documentent l'amélioration et/ou la diminution des départs.

7. Quels obstacles, défis et barrière avez-vous rencontrés concernant la conception et la mise en œuvre des systèmes de compensation de la PCIME-C?

8. Selon-vous et d'après l'expérience de votre organisation en matière de compensation des ASC, quels sont les principaux enseignements et les bonnes pratiques identifiées au fil du temps ?

9. Comment définiriez-vous la durabilité en termes de systèmes de compensation financière de la PCIME-C ?

10. Quels mécanismes ou stratégies utilise votre organisation en tant que voie durable de compensation ?

11. Quel impact a eu la pandémie de COVID-19 sur la politique de votre organisation en matière de compensation des ASC travaillant sur la PCIME-C/PECADOM ?
    Sonder pour: une réduction de la compensation due à la pandémie de COVID-19

12. Comment envisagez-vous l'avenir de la santé communautaire, à savoir de la PCIME C/PECADOM et des ASC dans le [PAYS] où vous travaillez ?

13. Pensez-vous qu'il y a autre chose d'important que je devrais savoir ?

14. Y a-t-il des documents que nous devrions inclure dans notre examen ?

15. Avez-vous des questions à me poser ? **Merci pour votre temps.**

Supplement to: Davis AL, Flomen L, Ahmed J, et al. Documenting community health worker compensation schemes and their perceived effectiveness in seven sub-Saharan African countries: a qualitative study. Glob Health Sci Pract. 2024;12(3):e2400008. https://doi.org/10.9745/GHSP-D-24-00008

Questions sur le pays au niveau national [Ministère de la santé, des finances, ou autre ministère pertinent]

Guide d'entretien du Ministère de la santé // Système de compensation financière des ASC – Évaluation

**Date de l'entretien**	
**Nom du répondant**	
**Poste**	
**Organisation**	
**Catégorie du répondant**	
**Informations supplémentaires sur le répondant**	
**Nom de l'enquêteur**	
**Heure de début**	
**Heure de fin**	

Notes pour l'enquêteur :

- *Avant de poser les questions ci-dessous, donnez le contexte de l'évaluation et passez en revue le processus de consentement informé. Si le répondant a déjà signé et retourné un formulaire de consentement rempli, fournissez une rapide vue d'ensemble de l'objectif de l'évaluation et du formulaire de consentement.*
- *Demandez si vous avez le consentement pour enregistrer l'entretien. Dans le cas contraire, veillez à prendre des notes de qualité]*
- *Au bout de 45 minutes, informez le répondant du nombre de questions restantes et voyez si vous pouvez terminer les questions durant les 15 minutes restantes, ou demander plus de temps si nécessaire.*
- *Écoutez attentivement les réponses du répondant et ne posez que les questions pertinentes par rapport à son rôle et sa fonction (par ex., si le répondant n'est pas impliqué dans le développement de capacités, ces questions peuvent ne pas être pertinentes)*

COMMENCEZ À ENREGISTRER

Introduction

1. Pour commencer, j'aimerais que vous me parliez un peu de votre travail avec [*votre unité*]. Pouvez-vous me parler un peu de votre/vos programme(s), votre rôle, et de comment vous êtes impliqué dans la PCIME-C ou dans les mesures de compensation des ASC dans [votre pays] ?

Sonder pour : la confirmation du cadre du travailleur chargé de réaliser la PCIME-C en général et pour le paludisme, et quel(s) service(s) coordonne la PCIME-C?

2. Pouvez-vous décrire la politique de compensation des ASC en général et pour la PCIME-C ?
   Sonder pour : Ceci fait-il partie d'une politique concernant les ASC ou d'une politique plus large ? Quels types de compensation financière ou non financière le système de santé est-il responsable de fournir ? Cette compensation financière comprend-elle la PCIME-C ? La politique de compensation de la PCIME-C est-elle alignée avec les politiques nationales de salaire minimum ?

3. Veuillez décrire le rôle de l'implication de la communauté dans la compensation financière et non financière des ASC.
   Sonder pour : La communauté est-elle impliquée, pouvez-vous me donner quelques exemples concrets de l'implication de la communauté dans la compensation des ASC, non financière, en nature ou financière ?

	**Provenant de qui ? (Système de santé/ Communauté/Autre)**		
		**Dans la politique ?**	**En cours de mise en œuvre**
**Financière**			
Incitation en espèces pour les services rendus			
Paiement de salaire direct et régulier			
Indemnités/avantages pour le transport et la formation			
Récompenses financières basées sur les performances			
Accès à des microcrédits			
Autres (préciser)			
**Non financier**			
Confiance dans la communauté			
Fait partie des conseils locaux			
Autres (préciser)			
**En nature**			
Privilèges spéciaux, tels que l'accès gratuit à des services sociaux			
Produits, animaux, nourriture, cadeaux, etc.			
Équipement : vélos, parapluies, badges, uniformes, téléphones portables, etc.			
Autres (préciser)			

4. Quels obstacles, défis et barrière avez-vous rencontrés concernant la **conception** de la compensation financière de la PCIME-C? [POUR CEUX DIRECTEMENT IMPLIQUÉS DANS LA CONCEPTION] Quels défis/barrières connaissez-vous concernant la conception des politiques, systèmes et processus de compensation ?
   Sonder pour : Comment déterminez-vous qui reçoit une compensation ?

5. [SI LE PAYS A AUSSI UNE COMPENSATION NON FINANCIÈRE, DEMANDEZ...] Quels obstacles, défis et barrière avez-vous rencontrés concernant la **conception** de la compensation non financière de la PCIME-C?

6. Quels types d'obstacles, défis et barrières avez-vous rencontrés concernant la **mise en œuvre** de la politique de compensation financière ?

   Sonder pour : Systèmes d'information qui réalisent le suivi du nombre et de l'emplacement des ASC
   Sonder pour : Précisez les obstacles spécifiques de la compensation financière et de la compensation non financière
   Sonder pour : À quel niveau se présentent ces obstacles – au niveau national, régional, du district, local (centre de santé) ou de la communauté ?
   Sonder pour : Tout problème de régularité et d'homogénéité des paiements de la compensation
   Sonder pour : Ce en quoi la mise en œuvre diffère de la conception

7. Quels rôles les acteurs nationaux, infranationaux et communautaires jouent-ils dans la mise en œuvre de la politique de compensation financière ?

   Sonder pour : Qui est responsable de distribuer la compensation pour la PCIME-C à chaque niveau ?
   Sonder pour : Les différents niveaux sont-ils en contact entre eux pour faciliter la distribution ?
   Sonder pour : Quels types de mécanismes utilisent-ils pour coordonner la compensation et communiquer à son sujet ?

8. Selon vous, parmi les différents problèmes/barrières/obstacles, lesquels sont les plus importants et pourquoi ?

   Sonder pour : Des plus importants aux moins importants et pourquoi

9. Quel impact a eu la pandémie de COVID-19 sur le travail des ASC dans le cadre de la PCIME-C? Quel impact a-t-elle eu sur la distribution de la compensation financière ?

   Sonder pour : Quels types de défis ou adaptations de la mise en œuvre de la PCIME-C avez-vous constatés pendant la pandémie ?
   Sonder pour : Y a-t-il eu un impact spécifique sur la compensation ? Étaient-elles encore fournies ? Réduites ?

10. En pensant aux difficultés (obstacles, défis, barrières) dont nous venons de parler, quelles stratégies [PAYS] a-t-il utilisé pour les résoudre ?
   Sonder pour : Quelles stratégies (le cas échéant) ont été développées pour chaque obstacles (même si elles se chevauchent)
   Sonder pour : Dans quelle mesure chaque stratégie a-t-elle été efficace ? Comment avez-vous vérifié son efficacité ?

11. Selon vous, dans le contexte de la PCIME-C, quels aspects de la compensation financière et non financière a fonctionné le mieux ?

12. Quels enseignement peuvent être tirés de l'expérience de [PAYS] dans la mise en œuvre de la compensation financière et non financière de la PCIME-C?
    Sonder pour : ceux qui sont spécifiquement pertinents pour [PAYS]
    Sonder pour : Pour chacun, comment ont-ils orienté la mise en œuvre ou la politique ?

13. Comment définiriez-vous la durabilité en termes de compensation financière de la PCIME-C ?

14. Dans quelle mesure pensez-vous que ces compensations financières de la PCIME-C sont durables ?
    Sonder pour : Qu'est-ce qui semble durable et pourquoi ?
    Sonder pour : Quelles sont vos préoccupations quant à la durabilité ?

15. Selon vous, dans quelle mesure les bonnes pratiques de compensation financière et non financière de la PCIME-C de [PAYS] sont pertinentes pour d'autres pays ?
    Sonder pour : Pourquoi ou pourquoi pas ?
    Sondez pour : la compensation financière et la compensation non financière

16. Existe-t-il des aspects du système de compensation financières qui doivent rester identiques pour être envisageable/durables dans le contexte d'un autre pays ?
    Sonder pour : Qu'est-ce qui peut être modifié sans perdre l'intégrité de la stratégie ?

17. Comment la compensation financière de la PCIME-Cvarie-t-elle de celle dans d'autres activités du secteur de la santé ?
    Sonder pour : Les stratégies et les enseignements tirés pourraient-ils être appliqués à des activités basées sur la communauté ?

18. Pensez-vous qu'il y a autre chose d'important que je devrais savoir ?

19. Y a-t-il des documents que nous devrions inclure dans notre examen ?

20. Avez-vous des questions à me poser ?

Merci pour votre temps.

Supplement to: Davis AL, Flomen L, Ahmed J, et al. Documenting community health worker compensation schemes and their perceived effectiveness in seven sub-Saharan African countries: a qualitative study. Glob Health Sci Pract. 2024;12(3):e2400008. https://doi.org/10.9745/GHSP-D-24-00008

Questionnaire pays au niveau sous-national [Acteurs régionaux et des districts qui soutiennent la compensation financière de la PCIME-C par les ASC]

Guide d'entretien au niveau sous-national // Système de compensation financière des ASC – Évaluation

**Date de l'entretien**	
**Nom du répondant**	
**Poste**	
**Organisation**	
**Catégorie du répondant**	
**Informations supplémentaires sur le répondant**	
**Nom de l'enquêteur**	
**Heure de début**	
**Heure de fin**	

Notes pour l'enquêteur :

- *Avant de poser les questions ci-dessous, donnez le contexte de l'évaluation et passez en revue le processus de consentement informé. Si le répondant a déjà signé et retourné un formulaire de consentement rempli, fournissez une rapide vue d'ensemble de l'objectif de l'évaluation et du formulaire de consentement.*
- *Demandez si vous avez le consentement pour enregistrer l'entretien. Dans le cas contraire, veillez à prendre des notes de qualité]*
- *Au bout de 45 minutes, informez le répondant du nombre de questions restantes et voyez si vous pouvez terminer les questions durant les 15 minutes restantes, ou demander plus de temps si nécessaire.*
- *Écoutez attentivement les réponses du répondant et ne posez que les questions pertinentes par rapport à son rôle et sa fonction (par ex., si le répondant n'est pas impliqué dans le développement de capacités, ces questions peuvent ne pas être pertinentes)*

COMMENCEZ À ENREGISTRER

Introduction

1. Pour commencer, j'aimerais que vous me parliez un peu de votre travail avec [*votre unité*]. Pouvez-vous me parler un peu de votre/vos programme(s), votre rôle, et de comment vous êtes impliqué dans la PCIME-C ou dans les mesures de compensation financière et non financière des ASC dans [votre pays] ?
   Sonder pour : la confirmation du cadre du travailleur chargé de réaliser la PCIME-C en général et pour le paludisme, et quel(s) service(s) coordonne la PCIME-C ?

2. Pouvez-vous décrire la politique de compensation financière des ASC, en particulier pour la PCIME-C?
    a. *Sonder pour :* Ceci fait-il partie d'une politique concernant les ASC ou d'une politique plus large ?
    b. *Sonder pour :* Quels types de compensation le système de santé est-il responsable de fournir ? Ces compensations comprennent-elles la PCIME-C?

3. Veuillez décrire le rôle de l'implication de la communauté dans la compensation financière et non financière des ASC.
    a. *Sonder pour :* La communauté est-elle impliquée, pouvez-vous me donner quelques exemples concrets de l'implication de la communauté dans la compensation des ASC, non financière, en nature ou financière ?
    b. *Sonder pour :* Si un exemple concret est fourni : Comment vous assurez-vous que ces exemples/compensations sont réellement mis en œuvre ?

	**Provenant de qui ? (Système de santé/Communauté/Autre)**		
		**Dans la politique ?**	**En cours de mise en œuvre**
**Financière**			
Incitation en espèces pour les services rendus			
Paiement de salaire direct et régulier			
Indemnités/avantages pour le transport et la formation			
Récompenses financières basées sur les performances			
Accès à des microcrédits			
Autres (préciser)			
**Non financier**			
Confiance dans la communauté			
Fait partie des conseils locaux			
Autres (préciser)			
**En nature**			
Privilèges spéciaux, tels que l'accès gratuit à des services sociaux			
Produits, animaux, nourriture, cadeaux, etc.			
Équipement : vélos, parapluies, badges, uniformes, téléphones portables, etc.			
Autres (préciser)			

4. Quels obstacles, défis et barrières avez-vous rencontrés concernant la mise en œuvre de la compensation financière et non financière de la PCIME-C telle que décrite dans la politique

à votre niveau ? Quels défis/barrières connaissez-vous concernant la mise en œuvre de la politique, des système et des processus de compensation financière et non financière ?

   a. *Sonder pour :* Comment déterminez-vous qui reçoit une compensation ?

5. Quels types d'obstacles et défis avez-vous rencontrés concernant la mise en œuvre de la politique de compensation financière ?
   a. *Sonder pour :* Précisez les obstacles spécifiques de la compensation financière et de la compensation non financière
   b. *Sonder pour :* À quel niveau se présentent ces obstacles – au niveau national, régional, du district, local (centre de santé) ou de la communauté ?
   c. *Sonder pour :* Tout problème de régularité et d'homogénéité des paiements de la compensation financière

6. Quels sont les responsabilités et les rôles joués à votre niveau dans la mise en œuvre de la politique de compensation financière ?
   a. *Sonder pour :* À votre niveau, qui est responsable de distribuer la compensation pour la PCIME-C?
   b. *Sonder pour : Votre niveau est-il en contact avec les niveaux supérieur et inférieur pour faciliter la distribution ?*
   c. *Sonder pour :* Quels types de mécanismes utilisent-ils pour coordonner la compensation et communiquer à son sujet ?

7. Selon vous, parmi les différents problèmes/barrières/obstacles, lesquels sont les plus importants ?
   a. Sonder pour : Des plus importants aux moins importants et pourquoi

8. Quel impact a eu la pandémie de COVID-19 sur le travail des ASC dans le cadre de la PCIME-C? Quel impact a-t-elle eu sur la distribution de la compensation financière ?
   a. Sonder pour : Quels types de défis ou adaptations de la mise en œuvre de la PCIME-C avez-vous constatés pendant la pandémie ?
   b. Sonder pour : Y a-t-il eu un impact spécifique sur la compensation ? Étaient-elles encore fournies ? Réduites ?

9. En pensant aux difficultés (obstacles, défis, barrières) dont nous venons de parler, quelles stratégies [PAYS] a-t-il utilisé pour les résoudre ?
   a. *Sonder pour :* Quelles stratégies (le cas échéant) ont été développées pour chaque obstacles (même si elles se chevauchent)
   b. *Sonder pour* : Dans quelle mesure chaque stratégie a-t-elle été efficace ? Comment avez-vous vérifié son efficacité ?

10. Selon vous, dans le contexte de la PCIME-C, quels aspects de la compensation financière et non financière a fonctionné le mieux ?

11. Quels enseignement peuvent être tirés de l'expérience de [PAYS] dans la mise en œuvre de la compensation financière de la PCIME-C ?
    a. *Sonder pour :* ceux qui sont spécifiquement pertinents pour [PAYS]
    b. *Sonder pour* : Pour chacun, comment ont-ils orienté la mise en œuvre ou la politique ?

12. Comment définissez-vous la durabilité en termes de compensation financière et non financière de la PCIME-C?

13. Dans quelle mesure pensez-vous que ces compensations financières de la PCIME-C sont durables ?
    a. *Sonder pour :* Qu'est-ce qui semble durable et pourquoi ?
    b. Sonder pour : Quelles sont vos préoccupations quant à la durabilité ?

14. Comment la compensation financière de la PCIME-Cvarie-t-elle de celle dans d'autres activités du secteur de la santé ?
    a. *Sonder pour : Connaissez-vous d'autres systèmes de compensation dans d'autres secteurs de la santé à votre niveau ?* Ces stratégies et les enseignements tirés pourraient-ils être appliqués à des activités basées sur la communauté ?

15. Pensez-vous qu'il y a autre chose d'important que je devrais savoir ?

16. Y a-t-il des documents que nous devrions inclure dans notre examen ?
17. Avez-vous des questions à me poser ?

Merci pour votre temps.

Questionnaire pour les ASC

Guide d'entretien au niveau des ASC // Système de compensation financière des ASC – Évaluation

**Date de l'entretien**	
**Nom du répondant**	
**Poste**	
**Organisation/District/Centre de santé**	
**Catégorie du répondant**	
**Informations supplémentaires sur le répondant**	
**Nom de l'enquêteur**	
**Heure de début**	
**Heure de fin**	

Notes pour l'enquêteur :

- *Avant de poser les questions ci-dessous, donnez le contexte de l'évaluation et passez en revue le processus de consentement informé. Si le répondant a déjà signé et retourné un formulaire de consentement rempli, fournissez une rapide vue d'ensemble de l'objectif de l'évaluation et du formulaire de consentement.*
- *Demandez si vous avez le consentement pour enregistrer l'entretien. Dans le cas contraire, veillez à prendre des notes de qualité]*
- *Au bout de 45 minutes, informez le répondant du nombre de questions restantes et voyez si vous pouvez terminer les questions durant les 15 minutes restantes, ou demander plus de temps si nécessaire.*
- *Écoutez attentivement les réponses du répondant et ne posez que les questions pertinentes par rapport à son rôle et sa fonction (par ex., si le répondant n'est pas impliqué dans le développement de capacités, ces questions peuvent ne pas être pertinentes)*

COMMENCEZ À ENREGISTRER

Introduction

1. Pour commencer, j'aimerais que vous me parliez un peu de votre travail en tant qu'ASC. Pouvez-vous m'en dire un peu plus sur votre rôle et sur ce que vous faites ?
   a. *Sonder pour :* son rôle dans la PCIME-C et les autres activités qu'il réalise en tant qu'ASC
   b. *Sonder pour :* Depuis combien de temps êtes-vous ASC/impliqué dans les activités de santé communautaire ?

2. Combien de temps consacrez-vous chaque semaine aux activités de PCIME-C dans votre/vos communauté(s) en tant qu'ASC ?

3. Vous arrive-t-il de payer vous-même certains articles, que ce soit pour votre propre usage au travail ou à l'usage de la communauté/des patients ?
   Sonder pour : les frais de transport ou autres frais encourus

4. De quelle manière l'établissement de santé est-il impliqué ou vous soutient-il dans la PCIME-C ?
   a. *Sonder pour :* Viennent-ils vous voir accomplir votre travail ? Fournir du matériel ? Fournir des médicaments ? Superviser et fournir une formation ?

5. De quelle manière la communauté est-elle impliquée ou vous soutient-elle dans la PCIME-C ?
   a. *Sonder pour :* Vient-elle vous voir accomplir votre travail ? Vous aide-t-elle dans d'autres activités ? Fournir du matériel ? Fournir des médicaments ? Existe-t-il un comité de santé communautaire ? Quel est son rôle ?

6. Avez-vous un accord officiel avec le Ministère de la santé, le centre de santé ou la communauté ?
   a. Si oui, cet accord est-il documenté par écrit ? Que comprend cet accord ? Énoncé des travaux ? Description de l'emploi ? Niveau et conditions de compensation financière ? Demandez à le voir

7. Lesquelles de ces incitations recevez-vous actuellement, ou devriez-vous recevoir ? [L'enquêteur énumère individuellement chaque incitation et sonde pour chaque colonne du tableau]
   a. *Sonder pour :* si cela s'applique à lui en tant qu'ASC en général, spécifiquement pour la PCIME-C, ou si c'est différent selon le programme qu'il supporte
   b. *Sonder pour :* si l'ensemble des incitations a changé au cours du temps ou si les paiements sont réguliers au cours du temps

	**Provenant de qui ? (Système de santé/Communauté/Autre)**	**Devrait recevoir**		**Reçoit effectivement**		**Apprécie l'incitation (pour ceux qui la reçoivent)**
		**Combien**	**À quelle fréquence**	**Combien**	**À quelle fréquence**	
**Financière**						
Incitation en espèces pour les services rendus						
Paiement de salaire direct et régulier						
Indemnités/avantages pour le transport et la formation						

Récompenses financières basées sur les performances						
Accès à des microcrédits						
Autres (préciser)						
**Non financier**						
Confiance dans la communauté						
Fait partie des conseils locaux						
Autres (préciser)						
**En nature**						
Privilèges spéciaux, tels que l'accès gratuit à des services sociaux						
Produits, animaux, nourriture, cadeaux, etc.						
Équipement : vélos, parapluies, badges, uniformes, téléphones portables, etc.						
Autres (préciser)						

8. Parmi celles que vous recevez, lesquelles appréciez-vous le plus ?

9. Quel impact a eu la pandémie de COVID-19 sur votre travail dans la PCIME-Cet sur les incitations ?
   Sonder pour : Quels types de défis ou adaptations de la mise en œuvre de la PCIME-C avez-vous constatés pendant la pandémie ?

   Sonder pour : Y a-t-il eu un impact spécifique sur les incitations financières et autres ? Étaient-elles encore fournies ? Réduites ? Différées ?

10. Pensez-vous qu'il y a autre chose d'important que je devrais savoir ?

11. Y a-t-il des documents que nous devrions inclure dans notre examen ?

12. Avez-vous des questions à me poser ?

Merci pour votre temps.

Supplement to: Davis AL, Flomen L, Ahmed J, et al. Documenting community health worker compensation schemes and their perceived effectiveness in seven sub-Saharan African countries: a qualitative study. Glob Health Sci Pract. 2024;12(3):e2400008. https://doi.org/10.9745/GHSP-D-24-00008

Supplement 2. Codebook of Deductive Codes for Key Informant Interviews

1	**TITLE**	**DESCRIPTION**
2	**CHW CODEBOOK**	
3	Change_compensation	Respondent's description of any changes in the financial or non-financial compensation they have received overtime
4	CHW_OOPE	Respondent's description of out of pocket expenses related to their CHW activities
5	CHW_role	Respondent's description of their role as a CHW
6	CHW_time	Respondent's description of their time commitments for CHW activities
7	Community_engagement	Respondent's description of health center engagement in their activities, including any compensation/support
8	COVID_impact	Respondent's thoughts on if and how COVID-19 impacted their work and/or compensation as a CHW
9	Govt_engagement	Respondent's description of Govt engagement in their activities, including any compensation/support [any support perceived as coming from
10	HC_engagement	Respondent's description of health center engagement in their activities, including any compensation/support
11	NGO_engagement	Respondent's description of NGO engagement in their activities, including any compensation/support
12	Policy_vs_Practice	Respondent's description of any differences between what they are supposed to receive and what they do receive in terms of CHW
13	Type_non_financial_compensation	Respondent's description of any non financial compensation (including but not limited to materials, gifts, morale incentives)
14	Type_financial_compensation	Respondent's description of any financial compensation
15	Value_compensation	Respondent's description of how much and/or why they value different forms of financial or non-financial compensation
16	**NATIONAL CODEBOOK**	
17	Best Practices	Respondent's reflections on best practices in implementing CHW compensation models
18	Challenges_design	Respondent's thoughts on barriers, challenges, roadblocks in designing CHW financial and non-financial compensation
19	Challenges_implementation	Respondent's thoughts on barriers, challenges, roadblocks in implementing CHW financial and non-financial compensation
20	Community_engagement	Description of community engagement in CHW compensation in the country, including any role of health communitees
21	Coordination_mechanisms	Respondent's thoughts on CHW or community health coordination, including task forces or committees throughout the country
22	COVID_impact	Respondent's thoughts on how COVID-19 impacted their organization's policy or implementation plan for CHW compensation (iCCM/CCM)
23	Current role	Respondent's current role with their organization (program description, engagement with iCCM)
24	Description of existing CHW model(s)	Overall descriptions of the CHW model or models existing in the country
25	Different_health_compensation	Respondent's description on different health financial compensation models that exist in their countries of engagment or knowledge
26	Evidence	Respondent's description of any studies, reports, strategic documents that are evidence of CHW financial compensation models or outcomes
27	Laws_policy	Description of any relevant laws or policies shaping approaches to CHW compensation in the country
28	Lessons_Scale_up	Respondent's reflections on relevancy/transferability of lessons learned in scale-up for other countries/contexts
29	Lessons Learned	Respondent's reflections on lessons learned in implementing CHW compensation models
30	Non-engagement	Organization's reasons for not engaging/ implementing CHW financial compensation
31	Overview Compensation Model	Respondent's description of their organization's CHW compensation model
32	CBO_engagement	Description of CBO engagement in CHW compensation in the country
33	Govt_engagement	Description of Govt engagement in CHW compensation in the country
34	NGO_engagement	Description of NGO engagement in CHW compensation in the country
35	Performance	Respondent's thoughts on how CHW financial compensation relates to CHW performance or attrition
36	Previous role(s)	Respondent's previous work related to community health, iCCM and CHWs
37	Rationale	Respondent's thoughts on rationales for supporting CHW financial and non-financial compensation
38	Reason_compensation	Respondent's thoughts on why their organization engages in CHW financial compensation
39	Role_compensation	Respondent's role in CHW financial compensation (technical and financial assistance)
40	Salary_alignment	Respondent's thoughts of if the CHW if in line with the national minimum regulations on salaries in the country
41	Strategies_overcome challenges	Respondent's thoughts on strategies and approaches that have helped to overcome challenges in CHW compensation
42	Strategy_sustainable compensation	Respondent's description of their organizations strategies or mechanisms to support sustainable CHW financial compensation
43	Sustainability_assessment	Respondent's assessment of the sustainability of approaches to CHW compensation practiced in the country
44	Sustainability_definition	Respondent's definition of sustainability with regards to CHW financial compensation
45	Timeline_compensation	Organization's timeline in in CHW financial and non-financial (in kind or other incentives) compensation
46	Type_financial_compensation	Respondent's description of type of CHW financial (in kind or other incentives) compensation they are implementing
47	Type_non_financial_compensation	Respondent's decription of non-financial compensation (materials, moral incentives, community in-kind gifts etc)
48	**SUBNATIONAL CODEBOOK**	
49	Best Practices	Respondent's reflections on best practices in implementing CHW compensation models
50	Challenges_implementation	Respondent's thoughts on barriers, challenges, roadblocks in implementing CHW financial and non-financial compensation
51	Community_engagement	Description of community engagement in CHW compensation in the country, including any role of health committee
52	Coordination_mechanisms	Respondent's thoughts on CHW or community health coordination, including task forces or committees throughout the country
53	COVID_impact	Respondent's thoughts on how COVID-19 impacted their organization's policy or implementation plan for CHW compensation (iCCM/CCM)
54	Current role	Respondent's current role with their organization (program description, engagement with iCCM)
55	Description of existing CHW model(s)	Overall descriptions of the CHW model or models existing in the country
56	Evidence	Respondent's description of any studies, reports, strategic documents that are evidence of CHW financial compensation models or outcomes
57	Laws_policy	Description of any relevant laws or policies shaping approaches to CHW compensation in the country
58	Lessons_Scale_up	Respondent's reflections on relevancy/ transferability of lessons learned in scale-up for other countries/contexts
59	Lessons Learned	Respondent's reflections on lessons learned in implementing CHW compensation models
60	Overall Compensation Model	Respondent's description of their organization's CHW compensation model
61	CBO_engagement	Description of CBO engagement in CHW compensation
62	Govt_engagement	Description of Govt engagement in CHW compensation
63	NGO_engagement	Description of NGO engagement in CHW compensation
64	Performance	Respondent's thoughts on how CHW financial compensation relates to CHW performance or attrition
65	Policy_vs_Practice	Respondent's description of any differences between policy and practice in CHW compensation implementation
66	Rationale	Respondent's thoughts on rationales for supporting CHW financial and non-financial compensation
67	Role_compensation	Respondent's role in CHW financial compensation (technical and financial assistance)
68	Strategies_overcome challenges	Respondent's thoughts on strategies and approaches that have helped to overcome challenges in CHW compensation
69	Sustainability_assessment	Respondent's assessment of the sustainability of approaches to CHW compensation practiced in the country
70	Sustainability_definition	Respondent's definition of sustainability with regards to CHW financial compensation
71	Type_compensation	Respondent's description of type of CHW financial and non-financial (in kind or other incentives) compensation being provided
